# ASC- and caspase-8-dependent apoptotic pathway diverges from the NLRC4 inflammasome in macrophages

**DOI:** 10.1038/s41598-018-21998-3

**Published:** 2018-02-28

**Authors:** Bettina L. Lee, Kathleen M. Mirrashidi, Irma B. Stowe, Sarah K. Kummerfeld, Colin Watanabe, Benjamin Haley, Trinna L. Cuellar, Michael Reichelt, Nobuhiko Kayagaki

**Affiliations:** 1 Department of Physiological Chemistry, Genentech Inc., South San Francisco, California, USA; 2 Department of Bioinformatics, Genentech Inc., South San Francisco, California, USA; 3 Department of Molecular Biology, Genentech Inc., South San Francisco, California, USA; 4 Department of Pathology, Genentech Inc., South San Francisco, California, USA

## Abstract

The NLRC4 inflammasome recognizes bacterial flagellin and components of the type III secretion apparatus. NLRC4 stimulation leads to caspase-1 activation followed by a rapid lytic cell death known as pyroptosis. NLRC4 is linked to pathogen-free auto-inflammatory diseases, suggesting a role for NLRC4 in sterile inflammation. Here, we show that NLRC4 activates an alternative cell death program morphologically similar to apoptosis in caspase-1-deficient BMDMs. By performing an unbiased genome-wide CRISPR/Cas9 screen with subsequent validation studies in gene-targeted mice, we highlight a critical role for caspase-8 and ASC adaptor in an alternative apoptotic pathway downstream of NLRC4. Furthermore, caspase-1 catalytically dead knock-in (Casp1 C284A KI) BMDMs genetically segregate pyroptosis and apoptosis, and confirm that caspase-1 does not functionally compete with ASC for NLRC4 interactions. We show that NLRC4/caspase-8-mediated apoptotic cells eventually undergo plasma cell membrane damage *in vitro*, suggesting that this pathway can lead to secondary necrosis. Unexpectedly, we found that DFNA5/GSDME, a member of the pore-forming gasdermin family, is dispensable for the secondary necrosis that follows NLRC4-mediated apoptosis in macrophages. Together, our data confirm the existence of an alternative caspase-8 activation pathway diverging from the NLRC4 inflammasome in primary macrophages.

## Introduction

Inflammation is a beneficial host mechanism that protects against injury caused by harmful agents such as pathogens or environmental irritants, however, excessive or chronic inflammation can be detrimental and lead to a myriad of inflammatory diseases^1^. Macrophages are vital sentinels of the immune system, surveying their environment via innate immune receptors, which include membrane-bound Toll-like receptors (TLRs) and cytosolic NOD-like receptors (NLRs). These receptors function in tightly regulated responses against pathogen-associated molecular patterns (PAMPs) derived from microbes or damage associated molecular patterns (DAMPs) released as a result of cellular injury^1–4^. Activation of most TLRs can promote NF-κB signaling and lead to the induction of pro-inflammatory cytokines, such as TNFα and IL-6, while some cytosolic NLRs, as well as AIM2 and MEFV/PYRIN, act as sensors in multi-protein inflammasome complexes.

Inflammasome stimulation leads to the enzymatic activation of inflammatory caspases, such as caspase-1 or caspase-11 (caspase-4 and caspase-5 in humans) and initiates pyroptosis, a rapid inflammatory programmed cell death marked by membrane disruption and passive release of cytoplasmic contents^5,6^. Pyroptosis is thought to be a beneficial mechanism for host defense not only to initiate inflammation, but also to expose bacteria hiding in intracellular niches to the extracellular milieu where they can be engulfed and sterilized by neutrophils and phagocytes^7^. Furthermore, inflammasome activation leads to maturation and release of inflammatory cytokines, such as IL-1β and IL-18, which play a key role in recruiting neutrophils to sites of infection and promote inflammatory anti-bacterial immune responses^3,8^. Apoptosis, by contrast, is often regarded as a non-inflammatory ‘silent’ programmed cell death pathway, preserving plasma membrane integrity and mediated by pro-apoptotic caspases, including caspase-8 and caspase-9 initiators and their downstream caspase-3 executor^9^.

The NLRC4 inflammasome specifically recognizes the cytosolic presence of flagellin and components of the type-III secretion system (T3SS) from several bacterial strains including *Salmonella*, *Legionella* and *Pseudomonas*^10–14^. NAIP proteins determine ligand specificity for NLRC4 activation; more specifically, NAIP5 and NAIP6 sense flagellin, while NAIP1 and NAIP2 recognize T3SS inner needle or rod proteins, respectively^13,14^. Ligand recognition by NAIPs initiates NLRC4 oligomerization and recruitment of caspase-1 via caspase recruitment domain (CARD) interactions, resulting in caspase-1 activation^15,16^. Activated caspase-1 then cleaves gasdermin-D (GSDMD), releasing the pore-forming GSDMD N-terminal fragment (GSDMD-NT), which immediately inserts into the plasma membrane, causing osmotic cell lysis^17–22^. Caspase-1 also processes the pro-form of IL-1β, resulting in the maturation and release of IL-1β from the cell either through the GSDMD pore or the ruptured plasma cell membrane.

Dysregulated NLRC4 contributes to severe disease beyond the context of microbial infections. Human *NLRC4* gain-of-function mutations (H443P, T337A and V341A) are linked to severe autoinflammatory diseases termed NLRC4-MAS (NLRC4 macrophage activation syndrome) or SCAN4 (syndrome of enterocolitis and auto-inflammation associated with mutation in NLRC4)^23,24^. Current treatments for NLRC4-MAS/SCAN4 focus on blocking IL-1, however, some patients respond poorly to IL-1 blockade suggesting that targeting upstream mechanisms of cell death may be a more effective treatment option. NLRC4 has also been implicated in the development of neuroinflammation and ischemic brain injury in pathogen-free conditions^25,26^. Thus, we sought to identify mechanisms of NLRC4-mediated cell death in sterile bacteria-free conditions to gain invaluable insights into the etiology of NLRC4 mediated auto-inflammatory diseases. Several studies have linked NLRC4 to an alternative caspase-8-mediated cell death distinct from caspase-1-dependent pyroptosis in various cell types and conditions^27–30^. However, genetic evidence of an alternative NLRC4 mediated caspase-8 pathway in primary macrophages with bacterial infection free conditions has not been clearly studied.

In this study, we performed an unbiased CRISPR/Cas9 screen followed by genetic confirmation experiments in primary macrophages from gene-targeted mice to gain a better understanding of the mechanisms involved in NLRC4-mediated cell death. Importantly, we provide genetic data that highlight the critical roles of ASC and apoptotic initiator caspase-8 in an alternative caspase-1-independent NLRC4-mediated cell death.

## Results

### NLRC4-mediated cell death occurs independently of caspase-1 in macrophages

**Figure 1:**
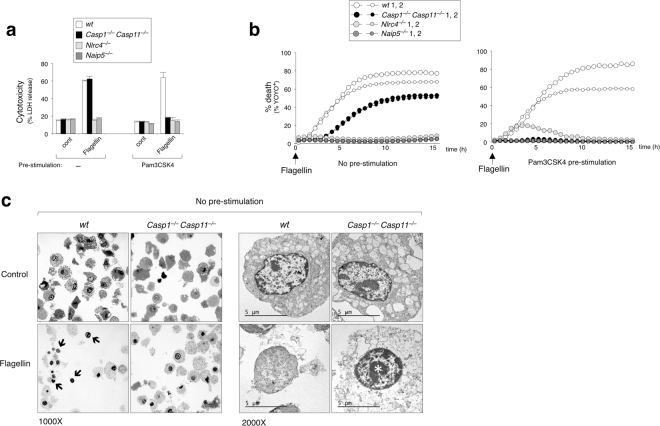
NLRC4 activates a caspase-1-independent cell death pathway in absence of TLR signaling. (**a**–**c**) BMDMs with or without Pam3CSK4 (1 μg ml^−1^) pre-stimulation were electroporated with flagellin (0.5 μg ml^−1^). (**a**) LDH release measured after 16 h. Data is represented as mean ± SD; n = 3. (**b**) % YOYO-1 positive BMDMs from two mice/genotype after flagellin electroporation. Live cell images taken every 30 min up to 16 h. (**c**) Transmission electron microscopy BMDMs 6 h after flagellin electroporation. Representative images captured at 1000× magnification or 2000× magnification. Black arrows (→) indicate free nuclei. White asterisks (*) indicate chromatin condensation.

To focus solely on NLRC4 inflammasome activation in macrophages, we delivered ultra-purified flagellin into the cytosol of bone marrow derived macrophages (BMDMs) by electroporation. Consistent with previous reports^10–12^, flagellin-triggered cell death measured by lactate dehydrogenase (LDH) release was fully dependent on NLRC4 and NAIP5 (Fig. 1a no pre-stimulation). Interestingly, *Casp1*^−/−^*Casp11*^−/−^ BMDMs released LDH to levels equivalent to that of wild-type (wt) under no pre-stimulation conditions, suggesting that NLRC4 may engage in an alternative non-pyroptotic cell death signal in the absence of caspase-1 (and caspase-11). To better understand the kinetics of cell death, we performed live cell imaging in the presence of a cell-impermeable fluorescent DNA staining dye (YOYO-1) to identify dead or dying cells. We confirmed that *Casp1*^−/−^*Casp11*^−/−^ BMDMs died and became YOYO-1^+^ in response to flagellin, albeit with slower kinetics compared to wt (Fig. 1b no pre-stimulation). When BMDMs were pre-stimulated with a TLR2-agonist (Pam3CSK4) as a method to mimic the presence of bacteria, *Casp1*^−/−^*Casp11*^−/−^ BMDMs became resistant to NAIP5/NLRC4-mediated cell death (Fig. 1a,b Pam3CSK4 pre-stimulation). This implies that TLR2 signaling can somehow block a caspase-1-independent alternative death signal.

To examine possible differences in cell death morphology, we captured transmission electron microscopy images of flagellin-stimulated wt and *Casp1*^−/−^*Casp11*^−/−^ BMDMs at 6 h under no pre-stimulation conditions (Fig. 1c). Wt cells exhibited typical necrotic/pyroptotic morphologies, such as plasma membrane rupture and organelle disintegration. Notably, intact free nuclei were frequently observed only in wt cells (Fig. 1c). In contrast, *Casp1*^−/−^*Casp11*^−/−^ BMDMs displayed chromatin condensation and maintained a relatively intact plasma cell membrane, characteristic of apoptosis^31^. Collectively, these data indicate that in the absence of caspase-1, NAIP5/NLRC4 inflammasome has the potential to trigger an alternative death pathway leading to a slower apoptotic-like outcome in myeloid cells.

### A genome-wide CRISPR screen identifies ASC and caspase-8 in NAIP5/NLRC4–mediated caspase-1-independent cell death

**Figure 2:**
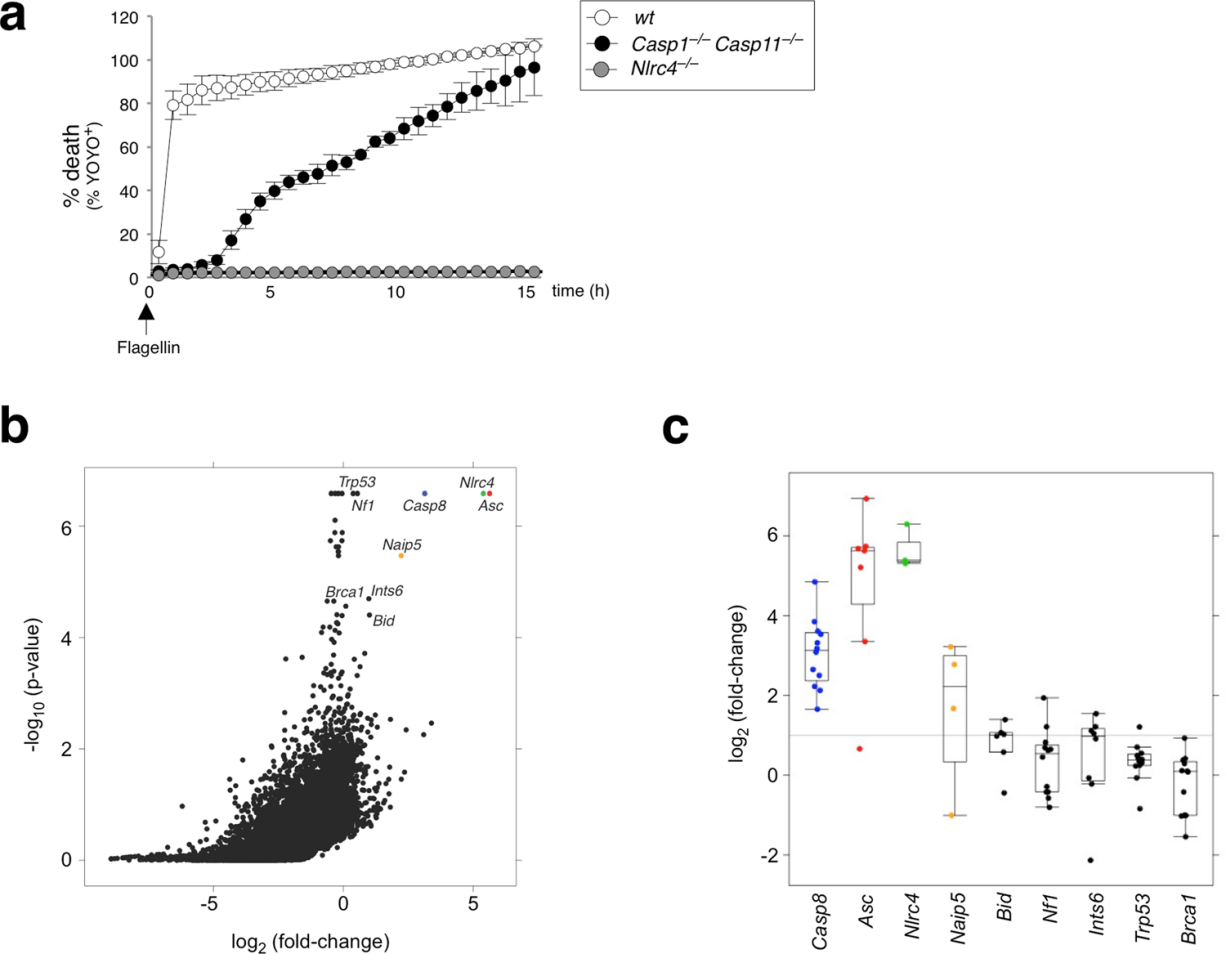
ASC and caspase-8 are identified through a genome-wide CRISPR/Cas9 screen for caspase-1-independent NLRC4-mediated cell death. (**a**) % YOYO-1 positive iMac cell lines from live imaging taken every hour up to 16 h after flagellin electroporation. Data is represented as mean ± SD; n = 3 images. (**b**) Scatter plot showing relative fold-change enrichment of genes (*x-axis*) with their corresponding enrichment *p* value (*y-axis*) from n = 3 biological replicates. Counts are log2 transformed and normalized using median scaling. Top scoring genes are highlighted. (**c**) Box-plot showing the distribution of individual sgRNA frequencies of flagellin- over control-treated populations, ordered left-to-right by increasing p-value.

CRISPR/Cas9 system is a powerful method to precisely delete genes of interest and the application of genome-wide CRISPR/Cas9 screens has been widely validated. To identify essential players involved in the alternative caspase-1-independent cell death pathway, we designed an unbiased genome-wide positive selection CRISPR/Cas9 screen. We first generated immortalized macrophage cells (iMac) from *Casp1*^−/−^*Casp11*^−/−^ mice and confirmed that NLRC4-mediated cell death in *Casp1*^−/−^*Casp11*^−/−^ iMacs proceeded with slower kinetics when compared to wt controls (Fig. 2a), which is consistent with our previous observations in BMDMs (Fig. 1b). *Casp1*^−/−^*Casp11*^−/−^ iMacs stably expressing Cas9 were infected with a lentivirus-based genome-wide single-guide RNA (sgRNA) expression library and subsequently electroporated with or without flagellin in absence of pre-stimulation. Surviving cells were isolated and the sgRNA sequences were compared between control and flagellin treated samples. As expected, *Nlrc4* and *Naip5* were among the top hits in our screen (Fig. 2b,c). Interestingly, while the adaptor ASC is known to be dispensable for NLRC4-induced pyroptosis^7^, *Asc* gRNA was significantly enriched in flagellin treated samples. Furthermore, apoptotic initiator *Casp8* was amongst the highest scoring genes, supporting our hypothesis that NLRC4-induced caspase-1-independent cell death is distinct from pyroptosis and is a caspase-8-dependent apoptotic cell death.

### Genetic evidence confirms role of *Asc* and *Casp8* in NLRC4-induced caspase-1-independent cell death

**Figure 3:**
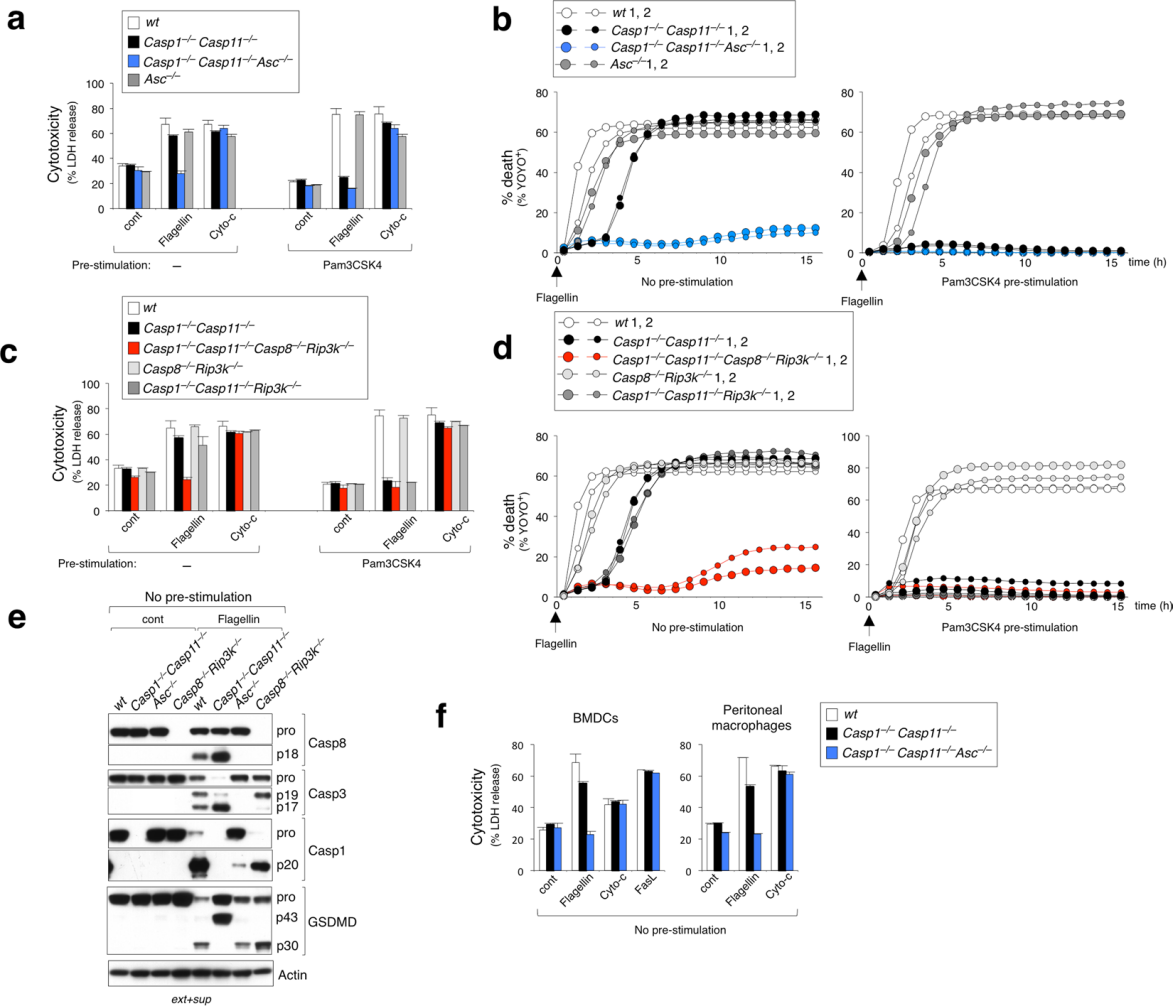
ASC and caspase-8 are required for caspase-1-independent NLRC4 activated cell death in BMDMs. (**a**–**d**) BMDMs with or without Pam3CSK4 (1 μg ml^−1^) pre-stimulation were electroporated with flagellin (0.5 μg ml^−1^) or cytochrome-c (50 μg ml^−1^). (**a**) and (**c**) LDH release after 16 h. Data is represented as mean ± SD; n = 3. (**b**) and (**d**) % YOYO-1 positive BMDMs from two mice/genotype after flagellin electroporation. Live cell images taken every 30 min up to 16 h. (**e**) Immunoblot of caspase-8, caspase-1, caspase-3 and GSDMD in combined cell extract (ext) and supernatant (sup) from BMDMs 3 hrs after flagellin electroporation (no pre-stimulation). Pro-forms (pro) and cleaved forms are represented in blots. (**f**) BMDCs and thioglycollate-elicited peritoneal macrophages with no pre-stimulation were electroporated with flagellin (0.5 μg ml^−1^), cytochrome-c (50 μg ml^−1^), or FasL (100 ng ml^−1^) and measured for LDH release after 16 h. Data is represented as mean ± SD; n = 3.

ASC and caspase-8 reportedly interact via the pyrin domain (PYD) on ASC and death effector domain (DED) on caspase-8 and were shown to co-localize to the same foci upon inflammasome activation^27,32^. Furthermore, another recent study demonstrated that targeting *Casp8* by CRISPR/sgRNA in immortalized macrophages abrogates NLRC4-induced apoptosis^29^. To test the involvement of ASC and caspase-8 in NLRC4-mediated cell death in primary macrophages, we generated *Asc*- or *Casp8* deficient *Casp1*^−/−^*Casp11*^−/−^ mice and determined their response to cytosolic flagellin. Unlike *Casp1*^−/−^*Casp11*^−/−^ BMDMs, triple-deficient *Casp1*^−/−^*Casp11*^−/−^*Asc*^−/−^ BMDMs were resistant to flagellin-induced cell death (Fig. 3a,b no pre-stimulation). Mice deficient in *Casp8* are embryonic lethal due to uncontrolled activation of necroptosis, however deletion of *Rip3k* can rescue this embryonic lethality^33,34^. Therefore, we generated *Casp1*^−/−^*Casp11*^−/−^*Casp8*^−/−^*Rip3k*^−/−^ mice to evaluate the role of caspase-8 in caspase-1-independent cell death. Similar to *Casp1*^−/−^*Casp11*^−/−^*Asc*^−/−^ BMDMs, *Casp8* deficiency in *Casp1*^−/−^*Casp11*^−/−^ BMDMs conferred resistance to NLRC4-mediated cell death (Fig. 3c,d no pre-stimulation). In contrast, all BMDMs tested responded normally to cytosolic cytochrome-c, a trigger for the Apaf-1/caspase-9-dependent intrinsic apoptosis pathway (Fig. 3a,c)^35^. Moreover, *Rip3k* deficiency did not alter NLRC4-induced caspase-1-independent cell death as *Casp1*^−/−^*Casp11*^−/−^*Rip3k*^−/−^ BMDMs were indistinguishable from *Casp1*^−/−^*Casp11*^−/−^ BMDMs (Fig. 3c,d no pre-stimulation). This genetic evidence demonstrates that ASC and caspase-8 are indispensable for the alternative caspase-1-independent death signal in primary macrophages, which is in line with previous studies^27–30^. In the presence of caspase-1, deficiency of *Asc* or *Casp8*/*Rip3k* alone did not affect the kinetics of NLRC4-mediated cell death (Fig. 3b,d no pre-stimulation), confirming that caspase-1-dependent pyroptosis is the dominant outcome in caspase-1-expressing BMDMs. As expected, pre-stimulation of BMDMs with Pam3CSK4 completely abrogated the caspase-1-independent ASC/caspase-8-dependent cell death response to NLRC4 activation (Fig. 3a–d Pam3CSK4 pre-stimulation).

We next sought to biochemically examine the downstream consequences of NLRC4 activation in the absence of TLR pre-stimulation. Upon flagellin recognition by NLRC4, caspase-8 is activated in both wt and *Casp1*^−/−^*Casp11*^−/−^ BMDMs, as demonstrated by the appearance of the cleaved large catalytic unit (p18) of caspase-8 (Fig. 3e). This indicates that NLRC4-mediated caspase-8 activation is a physiological event that can occur even in the presence of caspase-1 (wt cells). Substantially less cleaved caspase-8 was detected in wt cells when compared to *Casp1*^−/−^*Casp11*^−/−^ BMDMs. One possible explanation may be due to timing of cell death, as a majority of wt cells have undergone rapid pyroptotic cell death while *Casp1*^−/−^*Casp11*^−/−^ cells have additional time to cleave caspase-8 before undergoing apoptotic cell death. Strikingly, caspase-8 cleavage was completely abrogated in *Asc*^−/−^ BMDMs, suggesting that ASC acts upstream to promote caspase-8 activation. Comparable levels of pyroptotic activation were observed in both wt and *Casp8*^−/−^*Rip3k*^−/−^ BMDMs as indicated by the appearance of cleaved caspase-1 (p20) and GSDMD-NT (p30) in response to flagellin. In contrast, *Asc*^−/−^ BMDMs exhibited reduced caspase-1 p20 induction, but remarkably, generated normal levels of GSDMD-NT p30. Given the observation that *Asc*^−/−^ BMDMs succumb to NLRC4-mediated caspase-1-dependent pyroptosis with similar kinetics as wt control cells (Fig. 3c,d no pre-stimulation), we hypothesized that only a small amount of cleaved caspase-1 is sufficient to generate active GSDMD-NT p30 and initiate pyroptosis. However, we cannot rule out the possibility that unprocessed pro-caspase-1 could also efficiently cleave GSDMD to induce pyroptosis in *Asc*^−/−^ BMDMs^36^. Notably, *Casp1*^−/−^*Casp11*^−/−^ BMDMs generated an aberrant 43 kDa inactive GSDMD C-terminal fragment (GSDMD-CT p43) rather than the active GSDMD-NT p30 (Fig. 3e). This is consistent with previous studies showing that inactive fragments, GSDMD-NT p10 and GSDMD-CT p43, are aberrantly generated by caspase-3 cleavage of GSDMD after the aspartic acid at position 87 (D87) in cells lacking caspase-1^37^. Accordingly, there was no indication of typical pyroptotic morphology upon NLRC4 activation in *Casp1*^−/−^*Casp11*^−/−^ BMDMs (Fig. 1c), confirming that aberrantly cleaved GSDMD fragments lack sufficient pore forming activity^37^. Both caspase-1 and caspase-8 can cleave caspase-3, an executioner caspase of apoptosis^9,38^. As expected, flagellin stimulation induced processed caspase-3 in wt, *Casp1*^−/−^*Casp11*^−/−^ and *Casp8*^−/−^*Rip3k*^−/−^ BMDM. However, *Asc*^−/−^ BMDMs exhibited impaired caspase-3 processing, consistent with the attenuation of caspase-1 and -8 activation in these cells (Fig. 3e).

To determine whether other myeloid cells responded similarly to BMDMs, we tested bone marrow derived dendritic cells (BMDCs) and thioglycollate elicited peritoneal macrophages from *Casp1*^−/−^*Casp11*^−/−^ mice. We found these cells also underwent NLRC4 activated caspase-1-independent cell death, which was markedly reduced in absence of *Asc* (Fig. 3f). Together, we provide genetic and biochemical data which strongly support the model where ASC acts upstream of caspase-8 activation in macrophages undergoing NLRC4-induced apoptotic cell death.

### NF-κB signaling abrogates NLRC4-induced caspase-8-mediated cell death pathway

**Figure 4:**
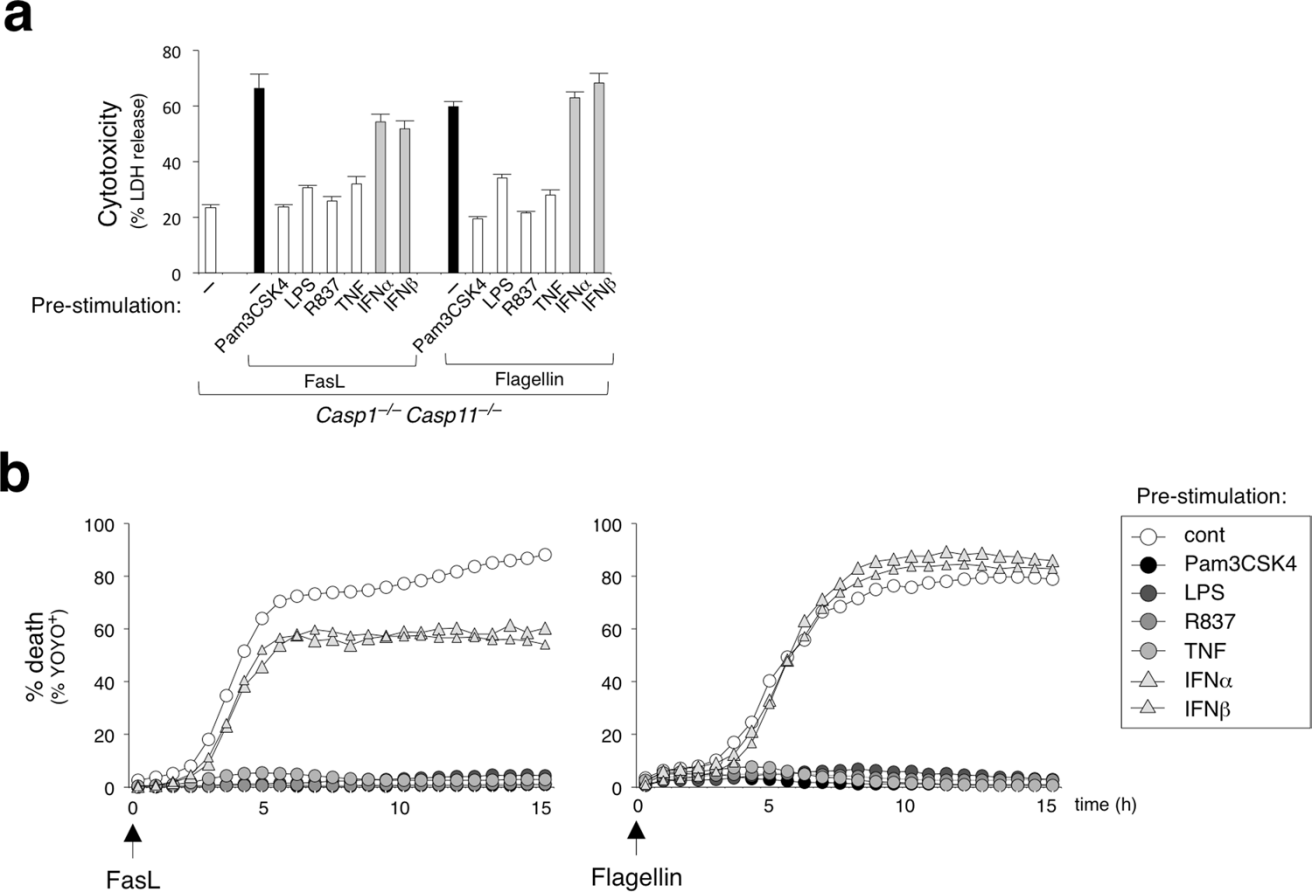
NF-κB signaling blocks NLRC4/caspase-8-dependent apoptosis pathway. *Casp1*^−/−^*Casp11*^−/−^ BMDMs were pre-stimulated for with or without various stimuli – Pam3CSK4 (1 μg ml^−1^), LPS (1 μg ml^−1^), R837 (2 μg ml^−1^), TNFα (100 ng ml^−1^), IFN-α (100 U ml^−1^), or IFN-β (100 U ml^−1^), then electroporated with flagellin (0.5 μg ml^−1^) or stimulated with FasL (100 ng ml^−1^). (**a**) LDH release after 16 h. Data is represented as mean ± SD; n = 3. (**b**) % YOYO-1 positive BMDMs from live cell imaging taken every 45 min for 16 h.

We have shown that pre-exposure to a TLR2 agonist disrupts the caspase-8-dependent death signal downstream of NLRC4 (Fig. 1a,b). It has been well-established that NF-κB signaling can trigger a transcriptional anti-apoptotic program^39^. Accordingly, we observed that pre-stimulation of BMDMs with various NF-κB activators including Pam3CSK4 (TLR2 agonist), LPS (TLR4 agonist), R837 (TLR7/8 agonist), TNFα, but not STAT-signaling activators IFN-α and IFN-β, blocked the known caspase-8-dependent Fas/FasL-mediated apoptosis pathway (Fig. 4a,b). We next investigated whether various NF-κB activators could also oppose NLRC4/caspase-8-dependent cell death in a similar manner. Indeed, the same NF-κB-activating treatments strongly attenuated NLRC4/caspase-8-dependent cell death in *Casp1*^−/−^*Casp11*^−/−^ BMDMs (Fig. 4a,b). These data support a model in which the NLRC4/caspase-8-mediated alternative apoptotic pathway proceeds dominantly in cells that (1) lack caspase-1 protein or perhaps where caspase-1 activation is somehow attenuated and (2) lack NF-κB activating signals or where signaling is blocked.

### Enzymatically inactive caspase-1 (C284A) fails to engage pyroptosis but does not abrogate caspase-8-dependent apoptosis downstream of NLRC4

**Figure 5:**
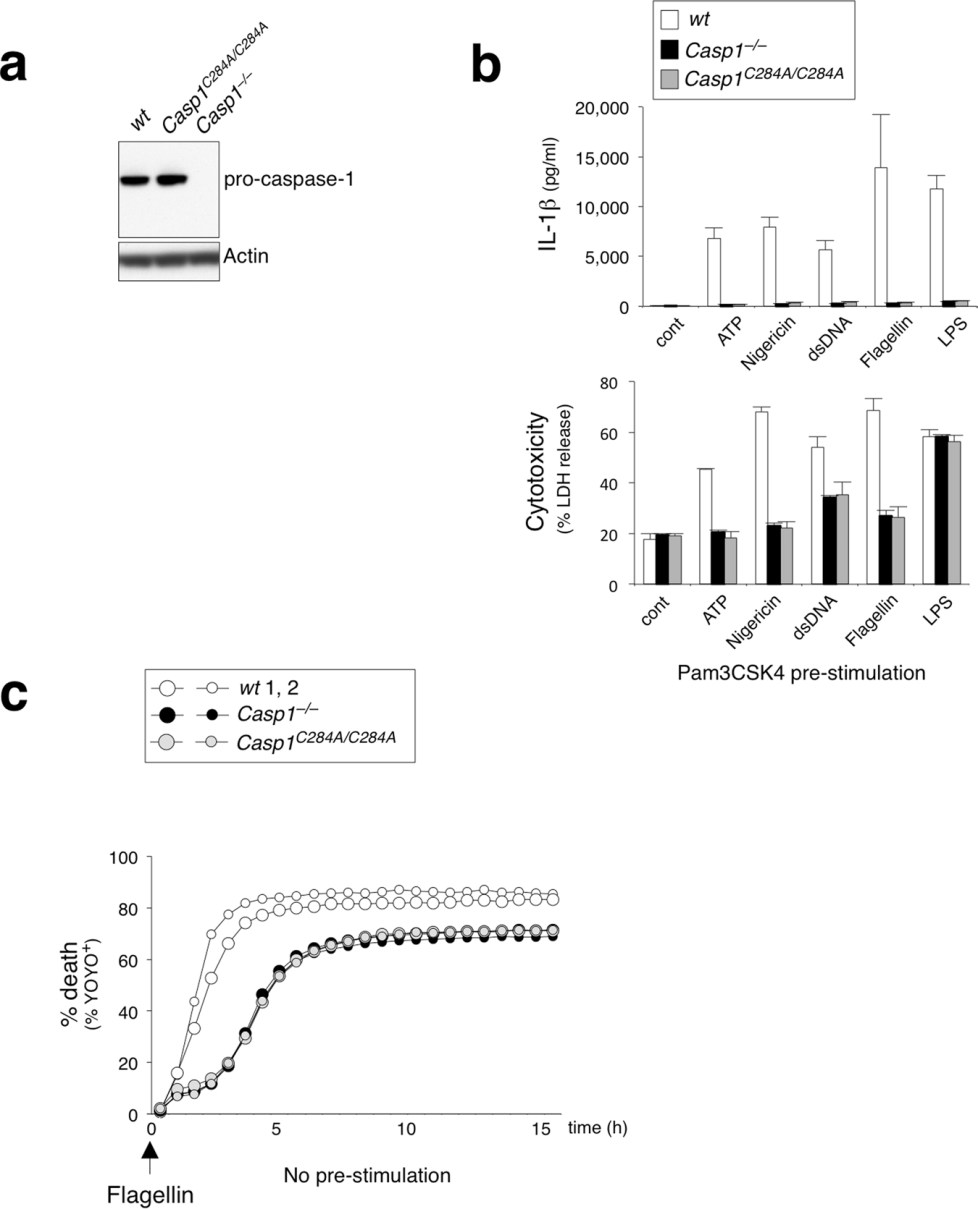
Caspase-1 does not compete with ASC for interaction with NLRC4 in enzymatically inactive *Casp1*^C284A/C284A^ BMDMs. (**a**) Immunoblot of pro-caspase-1 and actin from wt, *Casp1*^−/−^*, Casp1*^C284A/C284A^ BMDMs. (**b**) BMDMs with Pam3CSK4 (1 μg ml^−1^) pre-stimulation were electroporated with flagellin (0.5 μg ml^−1^). IL-1β release and LDH release measured after 16 h. Data is expressed as mean ± SD; n = 3. (**c**) % YOYO-1 positive BMDMs from two mice/genotype after stimulation. BMDMs with no pre-stimulation were electroporated with flagellin (0.5 μg ml^−1^). Live cell images taken every 45 min for 16 h.

Despite evidence of caspase-8 activation in wt cells (Fig. 3e), caspase-1-dependent pyroptosis is the primary mode of cell death in response to NLRC4 activation (Fig. 1). Our results have so far shown that NLRC4 activation skews toward an apoptotic outcome via ASC/caspase-8 in the absence of caspase-1 expression (*Casp1*^−/−^*Casp11*^−/−^, Fig. 1). One hypothesis to explain this observation would be that caspase-1 CARD preferentially interacts with the NLRC4-CARD, thereby blocking the availability of NLRC4 to interact with ASC-CARD. In the absence of caspase-1, NLRC4 would then be free to recruit ASC and proceed with the caspase-8-mediated apoptosis pathway. To test this competition hypothesis, we utilized *Casp1*^C284A/C284A^ knock-in mice harboring an enzymatically inactive mutation (C284A) to examine the consequence of full-length caspase-1 protein expression in absence of pyroptosis initiation. If this competition model were true, expression of caspase-1 C284A would block caspase-8-dependent death by competing with ASC-CARD for binding to NLRC4-CARD. We first confirmed that caspase-1 protein expression in *Casp1*^C284A/C284A^ BMDMs is comparable to that of wt (Fig. 5a). BMDMs derived from *Casp1*^C284A/C284A^ phenocopied their *Casp1*^−/−^ counterparts by exhibiting strong attenuation of pyroptosis and IL-1β release in response to multiple inflammasome stimuli, including ATP, Nigericin, as well as, intracellular dsDNA, flagellin and LPS, following Pam3CSK4 pre-stimulation (Fig. 5b), thus demonstrating that caspase-1 enzymatic activity plays an essential and non-redundant role in inflammasome signaling. However, contradicting the competition hypothesis, *Casp1*^C284A/C284A^ BMDMs continued to undergo caspase-8-mediated apoptosis with a similar time course as *Casp1*^−/−^ BMDMs in response to flagellin under no pre-stimulation conditions (Fig. 5c). Therefore, ASC/caspase-8 recruitment to NLRC4 is likely unperturbed in *Casp1*^C284A/C284A^ BMDMs, despite availability of the caspase-1 CARD for interaction. An alternative and more likely hypothesis may be that caspase-1/GSDMD-mediated pyroptosis is simply more rapid and potent, thereby prevailing over the delayed apoptotic signal.

### DFNA5 is dispensable for secondary necrosis following NLRC4/caspase-8-mediated apoptosis in macrophages

**Figure 6:**
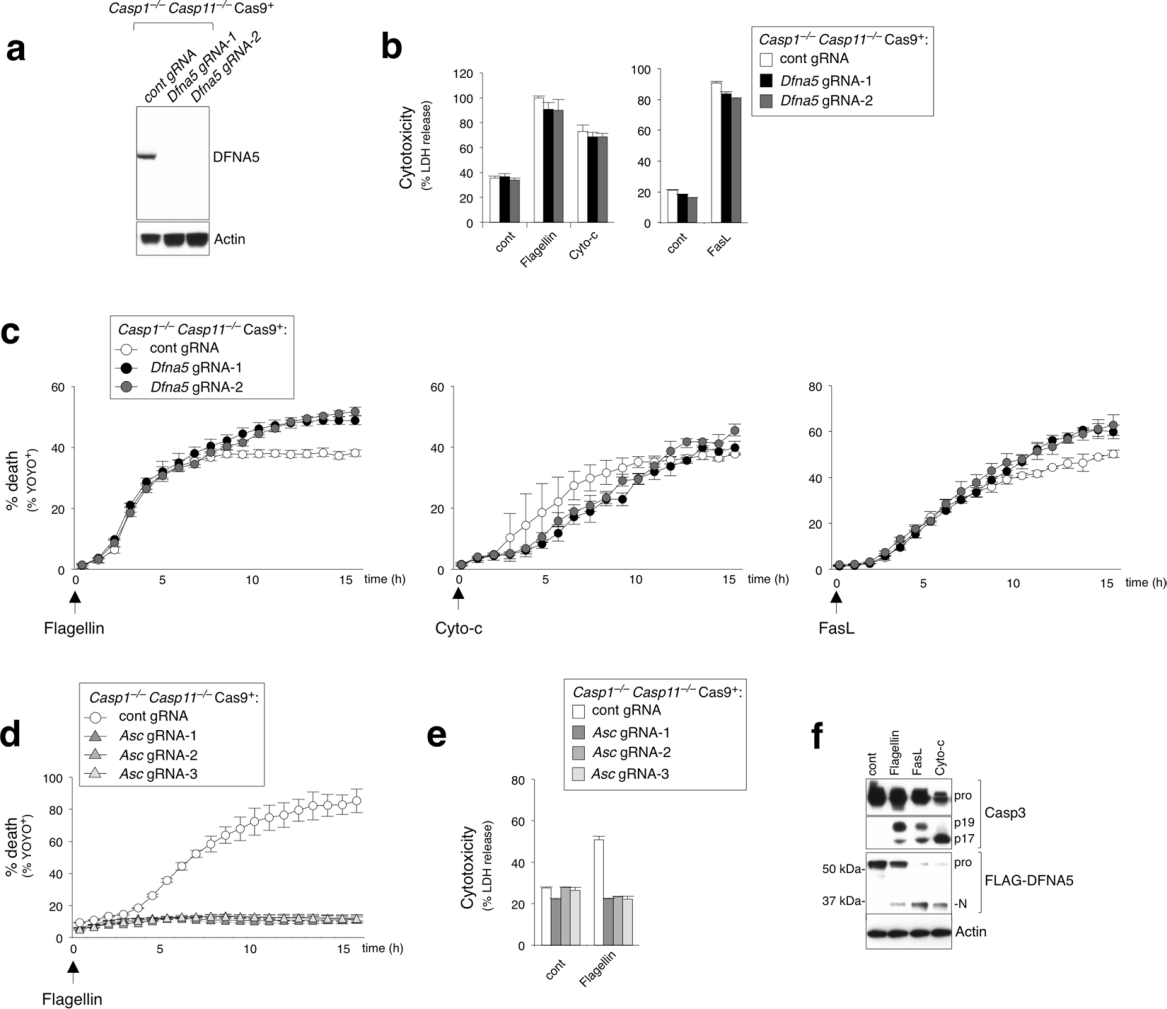
DFNA5 is dispensable for secondary necrosis after NLRC4/caspase-8-mediated cell death. (**a**) Immunoblot of mouse DFNA5 and actin from iMac whole cell lysates. (**b**–**e**) *Casp1*^−/−^*Casp11*^−/−^ iMac expressing *Dfna5*, *Asc* or luciferase (control) sgRNA were subjected to electroporation with flagellin (0.5 μg ml^−1^) and cytochrome-c (50 μg ml^−1^), or treated with FasL (100 ng ml^−1^). (**b**) and (**e**) LDH release after 16 h. Data is expressed as mean ± SD; n = 3. (**c**) and (**d**) % YOYO-1 positive iMacs from live cell imaging taken every hour for 16 h. Data is expressed as mean ± SD; n = 3. (**f**) Immunoblot for FLAG-tagged DFNA5 (FLAG antibody) and caspase-3 in combined cell extract and supernatant from FLAG-tagged DFNA5 expressing iMacs 3 hrs after electroporation with flagellin (0.5 μg) and cytochrome-c (25 μg), or treated with FasL (100 ng ml^−1^).

Apoptotic cells can undergo secondary necrosis if they are not effectively cleared by phagocytic cells^40^. As shown earlier, *Casp1*^−/−^*Casp11*^−/−^ BMDMs undergo NLRC4-induced caspase-8-dependent apoptosis and eventually release LDH and stain with a cell membrane impermeable dye, both readouts of plasma membrane damage (Figs 1a–c and 3a–d). These data suggest that secondary necrosis may ensue after apoptosis initiation under these conditions *in vitro*. Caspase-8 initiates apoptotic cell death by activating caspase-3^31,41^. Consistently, we observed caspase-3 activation in wt and *Casp1*^−/−^*Casp11*^−/−^ BMDMs following flagellin stimulation as evidenced by the induction of processed caspase-3 (Fig. 3e). Two recent reports demonstrated that caspase-3 cleaves DFNA5/GSDME, a member of the pore-forming gasdermin superfamily, leading to plasma membrane damage and subsequent osmotic burst^42,43^. Thus, we interrogated *Casp1*^−/−^*Casp11*^−/−^ iMacs to determine whether DFNA5 contributed to secondary necrosis following NLRC4/caspase-8-mediated apoptosis. To do so, we generated *Dfna5* deficient *Casp1*^−/−^*Casp11*^−/−^ iMacs using CRISPR/sgRNA and confirmed that knockout of *Dfna5* by sgRNA completely abrogated DFNA5 protein expression (Fig. 6a). Deficiency of DFNA5 did not inhibit LDH release nor delay the kinetics of plasma membrane damage after flagellin stimulation when compared to control sgRNA cells (Fig. 6b,c), whereas disrupting *Asc* by CRISPR/sgRNA completely abrogated cell death in response to flagellin (Fig. 6d,e). In contrast to recent reports^42,43^, we observed that *Dfna5* deficient *Casp1*^−/−^*Casp11*^−/−^ iMacs still succumbed to plasma membrane damage like wt control cells in response to other known activators of caspase-3 pathway, including FasL (via caspase-8) and cytochrome-c (via caspase-9) (Fig. 6b,c). All stimulants were confirmed to activate caspase-3 and convert the pro-form of DFNA5 into a ~35 kDa processed N-terminal DFNA5 form (with FLAG tag), which corresponds to the active pore-forming DFNA5 N-terminal (1–270) fragment generated by caspase-3, as previously reported^42,43^ (Fig. 6f). These observations suggest that while DFNA5 can be activated by caspase-3, it may be redundant for secondary necrosis in macrophages under these conditions, therefore highlighting a distinct DFNA5-independent program for plasma membrane rupture.

## Discussion

Pyroptosis constitutes an essential aspect of anti-bacterial innate immune defense. Pyroptosis is a rapid, irreversible and suicidal host defense mechanism for discharging invading intracellular bacteria from the host cell^5,6^. In our present study, we show that NLRC4 is capable of engaging an alternative ASC- and caspase-8-dependent apoptotic pathway, distinct from pyroptosis. NLRC4 has previously been implicated in a caspase-8-driven cell death pathway in several other studies. For example, ASC and caspase-8 in macrophages infected with *Salmonella Typhimurium* were shown to be co-recruited to the NLRC4 inflammasome and polymerize in the ‘ASC speck’^27^. Furthermore, in lung epithelial cell lines that lack caspase-1, forced expression of NLRC4 resulted in caspase-8 activation^28^. More recently, Rauch *et al*. provided genetic evidence that activation of NLRC4 by flagellin resulted in intestinal epithelial cells (IECs) expulsion activity but not lytic cell death in the absence of caspase-1 and this pathway required caspase-8 and ASC^29^. Another recent study using CRISPR/sgRNA showed that targeting *Casp8* in immortalized macrophages attenuated NLRC4-induced apoptosis^30^. Our unbiased CRISPR screening approach identified caspase-8 and ASC as non-redundant factors in NLRC4-mediated apoptotic cell death, which we corroborated with genetic evidence in BMDMs derived from gene-targeted mice to confirm the essential role of caspase-8 and ASC. The model of caspase-8-mediated apoptotic signal downstream of NLRC4/ASC is also in line with two recent independent reports^44,45^ that were published during revision of our manuscript.

Unlike other inflammasome sensors such as NLRP3 or AIM2, NLRC4 contains a CARD, which directly interacts with caspase-1 and negates the necessity for ASC as an adaptor protein for interaction with caspase-1^11^. However, we observed that ASC is necessary for NLRC4-mediated caspase-8 activation (Fig. 3e). Current literature supports the model in which ASC interacts with NLRC4 through CARD-CARD interactions and the ASC PYD domain recruits caspase-8 through an unusual heterotypic domain interaction with caspase-8 DED^32^. The biochemical nature of the ASC/caspase-8 interaction after recruitment to NLRC4 remains to be determined.

NLRC4-induced apoptosis is most clearly highlighted in cells lacking caspase-1 activity (Figs 1 and 4). Based on our findings, it seems likely that the NLRC4/caspase-1 and NLRC4/ASC/caspase-8 arms are parallel signaling pathways originating from the same NAIP5/NLRC4 signaling platform. We show that caspase-1 does not functionally compete with the ASC/caspase-8 pathway, as BMDMs expressing an enzymatically inactive mutant form of caspase-1 (C284A), which cannot engage in pyroptosis but retains the ability to interact with NLRC4 via CARD interactions, continue to undergo ASC/caspase-8-mediated apoptosis. Therefore, we suggest that the apoptotic outcome is likely overpowered by the rapid and prominent induction of caspase-1/GSDMD-dependent pyroptosis^17,18^. Interestingly, once macrophages sense other bacterial PAMPs recognized by TLRs, the apoptotic cell death pathway can no longer proceed. One mechanism is likely through inhibition of caspase-8 by cFLIP, following TLR/NF-κB activation^45,46^. From a host defense standpoint in the context of an intracellular bacteria infection, initiating pyroptosis over apoptosis would be more beneficial as exposure of “eat-me” signals on apoptotic cells could facilitate engulfment of dying infected cells by phagocytes^47^. The risk of phagocytes engulfing bacteria within apopotic cells could present a second chance for bacteria to replicate inside phagocytic cells. Interestingly, caspase-8 activation was also observed in wt cells undergoing pyroptosis, however, the physiological ramification of this remains unknown. Beyond apoptosis induction, caspase-8 is reported to have non-apoptotic functions including activation of chemokine transcription^48,49^. It may be possible that caspase-8-mediated chemokine induction contributes to anti-bacterial responses by recruiting neutrophils.

*NLRC4* gain-of-function mutations are associated with pathogen-free auto-inflammatory diseases. NLRC4-MAS/SCAN4 patients develop serious auto-inflammation, recurrent fever, rash and enterocolitis^23,24^. The existence of an alternative NLRC4-mediated caspase-8-activating pathway in TLR signaling-free conditions may contribute to deleterious tissue-damaging apoptotic signals from gain-of-function *NLRC4* mutants in cell types that perhaps do not express caspase-1 or where caspase-1 activity is blocked. In this scenario, uncontrolled or excessive caspase-8-activation could contribute to inflammation if apoptotic cells are not efficiently cleared^50^. This concept will require further exploration.

Downstream of caspase-8, caspase-3 has been reported to directly cleave DFNA5/GSDME and release the pore-forming DFNA5 N-terminal fragment, which leads to osmotic burst of the cell and necrotic cell death termed secondary necrosis^42,43^. DFNA5 is proposed to play a non-redundant role in caspase-3-induced secondary necrosis in BMDMs^42^. However, despite observing DFNA5 cleavage (Fig. 6f), we found that *Dfna5* deficiency did not inhibit nor delay secondary necrosis in our cells (Fig. 6b,c), implying that there could be other DFNA5-independent additional mechanisms that play a dominant role in mediating plasma membrane damage in response to flagellin, FasL and cytochrome-c *in vitro*. Molecular details of DFNA5-independent secondary necrosis remains unclear thus far, however it is unlikely that GSDMD plays a role since caspase-3 can only aberrantly cleave GSDMD to create a short inactive NT fragment (Fig. 3e and ref.^37^). DFNA5-independent secondary necrosis may represent an interesting and novel inflammatory mode of cell death, which will require further exploration into its mechanistic details and implications in inflammatory conditions *in vivo*.

## Materials and Methods

### Ethics statement

The Genentech Institutional Animal Care and Use Committee approved all animal studies. All experiments were performed in accordance with related institutional guidelines.

### Mice

*Casp1*^−/−^*Casp11*^−/− ^^51^*, Casp1*^−/− ^^18^, *Casp8*^−/−^*Rip3k*^−/− ^^52^, *Nlrc4*^−/− ^^53^ and *Asc*^−/− ^^51^ mice on a C57BL/6 N background were described previously. *Casp1*^−/−^*Casp11*^−/−^ were bred to *Asc*^−/−^ mice or *Casp8*^−/−^*Rip3k*^−/−^*. Naip5*^−/−^ lacking exons 8–10 were generated at Genentech from gene-targeted C57BL/6 N Tac ES cells. C57BL/6 N mice were purchased from Charles River Laboratories. *Naip5*^−/−^ mice were genotyped with PCR primers (5′-CCTTCCCTGTCCTTCTGATTT; 5′-AGCCTGCTTCTTCTAACCTTATAG and 5′-CAGGACTTTGTGTATTGTGGATTT) yielding a 424 -bp wild-type DNA fragment and a 268-bp mutant DNA fragment. *Casp1*^C284A/C284A^ were generated at Genentech from gene-targeted C57BL/6 N Tac ES cells. *Casp1*^C284A/C284A^ mice were genotyped with PCR primers (5′-GGAGATGGTGAAAGAGGTGAAAGA and 5′-GTCTCAATGGGAATGCCCTGTTA) and probes (VIC/5′-ATTCAGGCATGCCGTGGAGG/MGBNFQ and FAM/5′-ATTCAGGCAGCCCGTGGAGG/MGBNFQ).

### Reagents and antibodies

Ultra-pure flagellin (*Psuedomonas aeruginosa*), Pam3CSK4, R837 (Imiquimod), ultra-pure LPS (*E. coli* O111:B4), Poly(dA:dT), Nigericin were purchased from Invivogen, Mega FasL from AdipoGen, IFN-α and IFN-β from PBL Assay Science, TNFα from Genentech, ATP and bovine cytochrome-c from Sigma, YOYO-1 dye from Thermofisher Scientific, Nuclear-ID DNA stain from Enzo Life Sciences. Antibodies used include: mouse caspase-1 (clone 4B4, Genentech), Gsdmd (17G2G9, Genentech), caspase-3 (Cell Signaling Technology), cleaved active caspase-3 (5A1E, Cell Signaling Technology), caspase-8 (1G12, Enzo), cleaved active caspase-8 (D5B2, Cell Signaling Technology), IL-1β (GTX74034, GeneTex) and actin (AC15, NOVUS) and DFNA5 rabbit polyclonal antibody was raised against ESDFVKYESKCENHKSGAIG peptide (mouse DFNA5 74–93 amino acid) and FLAG M2 antibody (Sigma).

### Primary cell culture and stimulations

Bone marrow cells were differentiated into macrophages in DMEM with 10% endotoxin-free fetal bovine serum (Omega Scientific) and 20% L929-conditioned medium for 5–6 days, then plated at ~1.0 × 10^6^ cells ml^−1^ with 100 ul in 96-well and cultured overnight. For bone marrow derived dendritic cells, bone marrow cells were differentiated for 5 days in RPMI with 10 with 10% endotoxin-free fetal bovine serum and 20 ng ml^−1^ GM-CSF (eBiosciences). For stimulations, cells were pre-stimulated with 1 μg ml^−1^ Pam3CSK4, 1 μg ml^−1^ LPS, 2 μg ml^−1^ R837, 100 ng ml^−1^ TNFα, 100 units ml^−1^ IFN-α, or 100 units ml^−1^ IFN-β for 5 h where indicated and then cultured in OPTI-MEM media (Invitrogen) with indicated stimulations, 100 ng ml^−1^ FasL, 5 mM ATP, 2 μg ml^−1^ dsDNA/Poly(dA:dT) plus 0.1% v/v Lipofectamine 2000 (Life Technologies), 5 μg ml^−1^ LPS plus 0.25% v/v FuGENE HD (Promega)^54^, Nigericin 5 μg ml^−1^ or subjected to electroporations, as indicated. For AMAXA electroporations, 500 ng ml^−1^ Flagellin or 50 μg ml^−1^ cytochrome-c were electroporated into ~5.0 × 10^6^ cells in OptiMEM media 24-well plates using the AMAXA 4D-Nucleofector system Y-unit (Lonza). For Neon electroporations, the Neon transfection system (Life Technologies) was used with 1720 Voltage, 10 Width, 2 Pulse settings and performed with 1 × 10^6^ cells plus Flagellin 0.5 μg or 25 μg cytochrome-c per electroporation condition and plated at 1 × 10^5^ cells per 96-well for imaging and assays.

### Cell death and cytokine measurements

LDH release was measured using CytoTox 96 Non-Radioactive Cytotoxicity Assay (Promega) according to manufacturer’s instructions. Data calculated as signal over max death signal (cells treated with 0.1% Triton). YOYO-1 (491/509) dye (at 200 nM final concentration) was added at the time of stimulation and scanned every 30 min–1 hr for at least 16 hours on the Essen BioScience IncuCyte ZOOM at 10× magnification, scanned in green channel. Nuclear-ID was added at the last time point, scanned in red channel. IncuCyte software was used to determine total number of dead YOYO^+^ cells and Nuclear-ID^+^ (live and dead). Percent death (or % YOYO^+^) was calculated as the number of YOYO^+^ cells divided by the total number of NuclearID^+^ positive cells. IL-1β was measured from cell culture supernatants by mouse IL-1β Tissue culture kit (Meso Scale Discovery).

### Immunoblotting

For immunoblotting (extract + supernatant), ~8.0 × 10^5^ cells were electroporated with flagellin by Neon electroporation, then incubated in 75 ul OptiMEM media for 3 hours. Supernatant was separated and cells were lysed in RIPA buffer (50 mM Tris-HCl pH 7.4, 150 mM NaCl, 1 mM EDTA, 1× Complete Protease Inhibitor (Roche), 1% Triton X-100, 0.1% SDS) then combined with supernatant with additional protease inhibitor. For all other immunoblots, 5.0 × 10^4^ cells were lysed in RIPA buffer and run as whole cell lysate. Full-length blots are presented in Supplementary Figure S1.

### Immortalized macrophages and CRISPR-Cas9 technology

Bone marrow cells from wt, *Casp1*^−/−^*Casp11*^−/−^ or *Nlrc4*^−/−^ were immortalized by ER-Hoxb8 (iMac) as described previously^55^. *Casp1*^−/−^*Casp11*^−/−^ iMacs were then retrovirally transduced with human codon optimized *Streptococcus pyogenes* Cas9 [cloned in pMX-GFP (CellBioLabs)] and sorted for GFP-positive cells. Single guide RNAs (sgRNA) were transduced into *Casp1*^−/−^*Casp11*^−/−^ Cas9^+^ iMac cell lines by lentiviral delivery with pLKO.1 vector (Sigma) followed by selection of sgRNA-expressing cells with Puromycin at 1 μg ml^−1^ (Life Technologies). For individual sgRNA studies, sgRNA expressing *Casp1*^−/−^*Casp11*^−/−^ Cas9^+^ iMac were incubated with L929 conditioned media for 5 days, then harvested for experiments following same protocol as BMDMs using Neon electroporation where indicated. *Dfna5* sgRNA-1 5′-AAGCTGCAACTTCTAAGTC-3′, *Dfna5* gRNA-2 5′-AAAGAAGAGATACTGGTGC-3′, *Asc* sgRNA-1 5′-TGACAGTGCAACTGCGAGA-3′, *Asc* sgRNA-2 5′- TGCAACTGCGAGAAGGCTA-3′, Asc sgRNA-3 5′-TATGGGCGCATCCCACGCG -3′, luciferase control (Luc) sgRNA 5′-GCATGCGAGAATCTCACGC-3′. For FLAG-DFNA5 studies, wt Cas9^+^ iMac expressing *Dfna5* gRNA-3 5′-GTGAGTACATCTTCCAGGG-3′ complemented with gRNA resistant 3xFLAG (N-terminus)-tagged mouse Dfna5 cDNA by lentiviral delivery in pLenti6.3 vector (ThermoFisher Scientific). Immunoblotting (extract + supernatant) was performed as described above after Neon electroporation with flagellin and cytochrome-c (as described above), or incubation with 100 ng ml^−1^ FasL, in 70 μl total volume.

### Genome-wide CRISPR-Cas9 Screen

For CRISPR sgRNA library screen, 155,164 guides targeting 19,884 mouse genes were selected by using a custom sgRNA design algorithm and the designated guides were cloned into a pooled lentiviral expression context by Cellecta, Inc. *Casp1*^−/−^*Casp11*^−/−^ Cas9^+^ iMac were infected with a virus library titer to achieve a MOI of 0.3, with sufficient cell numbers plated to obtain a screening depth of 200 cells per sgRNA. After 13 days post-selection, cells were subjected to Neon electroporation with or without flagellin. Genomic DNA isolation was performed using Puregene reagents (Qiagen). Next generation sequencing was performed on MiSeq system (Illumina) with 50 million sequences reads per sample. Screen hits were identified by MAGeCK computational analyses for sgRNA enrichment in flagellin compared to control treatment. Raw counts were filtered to remove low abundance guides using heuristic from the gCrisprTools package that is gRNAs with counts less than 1/16^th^ of the top 1000 guides for each sample. Counts are log2 transformed and normalized using median scaling. Guide and gene-level statistics comparing flagellin and control samples are calculated using the MAGeCK software with default parameters. Genes with adjusted p-values less than 0.05 and a log2 fold-change greater than 1 were selected for further investigation. Screen samples were performed in triplicate.

### Standard (morphology) Transmission Electron Microscopy of Cells

Flagellin 0.5 μg per 1 × 10^6^ cells was delivered into BMDMs by Neon electroporation and harvested at 6 hours. A total of 30E6 cells per sample were fixed in 1.0 ml of 1/2 Karnovsky’s fixative (2% paraformaldehyde, 2.5% glutaraldehyde in 0.1 M sodium cacodylate buffer, pH 7.2). Samples were post-fixed in 1% aqueous osmium tetroxide for 2 h, stained with 0.5% uranyl acetate for 1 h and then dehydrated through a series of ethanol (50%, 70%, 90%, 100%) followed by two propylene oxide washes. Samples were embedded in Eponate 12 (Ted Pella, Redding, CA). Curing of the samples was at 65 °C for two days. Semithin (300 nm) and ultrathin (80 nm) sections were obtained with an Ultracut microtome (Leica). The semithin sections were stained with Toluidine Blue and examined by bright field microscopy to obtain overviews of the population of cells. Bright field images were captured with an Axioplan microscope (Zeiss), an AxioCAM MRm digital camera (Zeiss) and oil immersion lenses Plan-Neofluar 40×/1.4 N.A and a Plan-Neofluar 100×/1.4 N.A (Zeiss). Ultrathin sections parallel to the T-Blue sections were collected on electron microscopy grids, counter stained with 0.2% lead citrate and examined in a JEOL JEM-1400 transmission electron microscope (TEM) at 80 kV. Digital images were captured with a GATAN Ultrascan 1000 CCD camera.

### Data Availability

The datasets generated during and/or analysed during the current study are available from the corresponding author on reasonable request.

## Electronic supplementary material

Supplementary Information
