# Supplementary material for: ASC- and caspase-8-dependent apoptotic pathway diverges from the NLRC4 inflammasome in macrophages
Source: Sci Rep. 2018 Feb 28;8:3788. doi: 10.1038/s41598-018-21998-3 (PMC5830643; doi:10.1038/s41598-018-21998-3)
Supplement: Supplementary file 1 — Supplementary Information [file 41598_2018_21998_MOESM1_ESM.doc]

**Supplementary Figures**

**ASC- and caspase-8-dependent apoptotic pathway diverges from the NLRC4 inflammasome in macrophages**

Bettina L. Lee^1^, Kathleen M. Mirrashidi^1^, Irma B. Stowe^1^, Sarah K. Kummerfeld^2^, Colin Watanabe^2^, Benjamin Haley^3^, Trinna L. Cuellar^3^, Michael Reichelt^4^, and Nobuhiko Kayagaki^1*^

^1^Department of Physiological Chemistry, Genentech Inc., South San Francisco, California, USA

^2^Department of Bioinformatics, Genentech Inc., South San Francisco, California, USA

^3^Department of Molecular Biology, Genentech Inc., South San Francisco, California, USA

^4^Department of Pathology, Genentech Inc., South San Francisco, California, USA

*Corresponding Author

Email: kayagaki.nobuhiko@gene.com


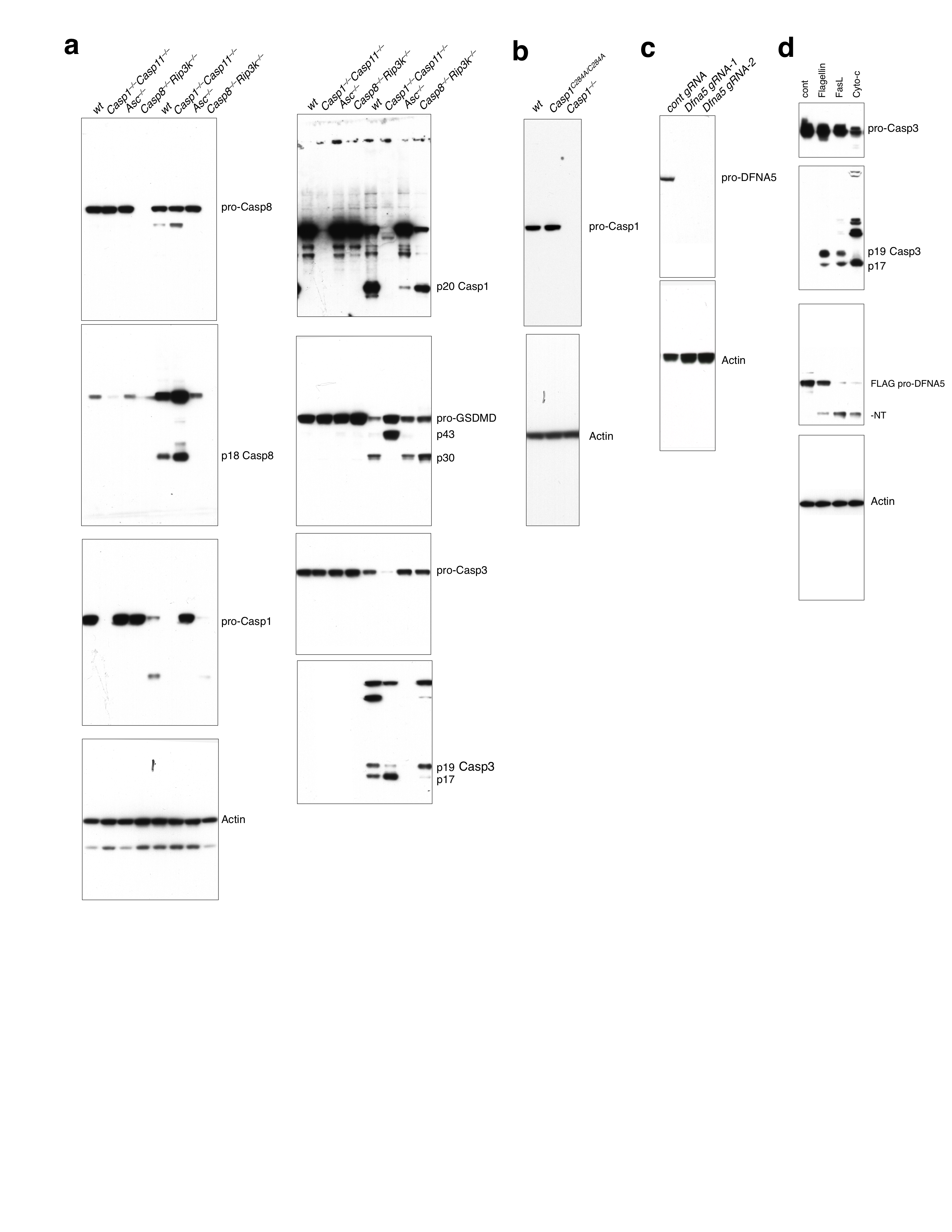


**Supplementary Figure S1**

Full-length immunoblots from main figures. **(a)** From Figure 3e, blots probed with caspase-8, caspase-1, GSDMD, caspase-3, and actin antibodies. **(b)** From Figure 5a, blots probed with caspase-1 and actin antibodies. **(c)** From Figure 6a, blots probed with DFNA5 and actin antibodies. **(d)** From Figure 6f, blots probed with caspase-3, FLAG, and actin antibodies.
